# Current Surgical Aspects of Palliative Treatment for Unresectable Pancreatic Cancer

**DOI:** 10.3390/cancers3010636

**Published:** 2011-02-11

**Authors:** Konstantinos Karapanos, Iakovos N. Nomikos

**Affiliations:** Department of Surgery (B′ Unit), “METAXA” Cancer Memorial Hospital, Piraeus, Greece; E-Mail: nomikosj@otenet.gr

**Keywords:** pancreatic cancer, locally advanced, unresectable, palliative care, surgery

## Abstract

Despite all improvements in both surgical and other conservative therapies, pancreatic cancer is steadily associated with a poor overall prognosis and remains a major cause of cancer mortality. Radical surgical resection has been established as the best chance these patients have for long-term survival. However, in most cases the disease has reached an incurable state at the time of diagnosis, mainly due to the silent clinical course at its early stages. The role of palliative surgery in locally advanced pancreatic cancer mainly involves patients who are found unresectable during open surgical exploration and consists of combined biliary and duodenal bypass procedures. Chemical splanchnicectomy is another modality that should also be applied intraoperatively with good results. There are no randomized controlled trials evaluating the outcomes of palliative pancreatic resection. Nevertheless, data from retrospective reports suggest that this practice, compared with bypass procedures, may lead to improved survival without increasing perioperative morbidity and mortality. All efforts at developing a more effective treatment for unresectable pancreatic cancer have been directed towards neoadjuvant and targeted therapies. The scenario of downstaging tumors in anticipation of a future oncological surgical resection has been advocated by trials combining gemcitabine with radiation therapy or with the tyrosine kinase inhibitor erlotinib, with promising early results.

## 1. Introduction

Pancreatic and periampullary neoplasms are the fifth leading cause of cancer-related death in the Western world [1,2]. Most of them are pancreatic adenocarcinomas, with a poor overall five-year survival, varying from 8% for stage I to 3% for stage IV [3]. It is noticeable that the survival of such patients has barely improved over the last years, despite all efforts at providing a more effective therapy [4,5]. In most cases (85%), the disease has already reached an incurable state at the time of diagnosis, due to extensive local disease or metastases, making an oncological resection feasible in only 13% of patients [3,6,7]. It is, therefore, obvious that accurate clinical staging of pancreatic cancer is mandatory, since it determines all consequent therapeutic steps.

According to the current American Joint Committee on Cancer (AJCC) TNM classification, localized resectable pancreatic cancer is represented by stages I and II, locally advanced cancer by stage III (T4, tumor involves the celiac axis or the superior mesenteric artery) and metastatic cancer by stage IV (M1, distant metastases present). Locally advanced pancreatic cancer, which is considered as unresectable primary tumor, is the main topic of this review. It must be noted that tumor involvement of the superior mesenteric vein and/or the portal vein alone, is classified as T3, thus resectable tumor stage II. In other words, the involvement of the celiac axis and/or the superior mesenteric artery is the key element to categorize a non-metastatic pancreatic cancer as resectable or not [8].

Historically, it has been questioned whether cure is possible at all in pancreatic cancer patients [4]. Today, there is consensus that surgical resection remains the best single chance these patients have for long-term survival [9]. Still, most patients are only eligible for palliative treatment at the time of diagnosis, and palliation of symptoms remains the major goal. The three most important symptoms that usually need to be treated in advanced pancreatic cancer are obstructive jaundice, gastric outlet obstruction and pain.

There are two different time points for establishing the respectability or not of a pancreatic adenocarcinoma: on preoperative workup and during laparotomy or staging laparoscopy. The optimal palliative treatment for the first group is generally considered nonsurgical, consisting mainly of endoscopic procedures (stenting) for biliary or duodenal obstruction, and percutaneous splanchnicectomy for pain relief. Palliative surgery is limited for the cases that failed nonsurgical management or presented significant complications from it, such as penetration, bleeding or stent occlusion. On the other hand, patients with pancreatic cancer that is proven unresectable at the time of the staging laparotomy, undergo palliative surgical therapy predominantly.

The aim of this article is to present as clear and practically as possible the role of surgery in the current status of palliative care for pancreatic cancer. Obstructive jaundice, gastric outlet obstruction, pain, palliative resection, laparoscopic palliation and the new era of neoadjuvant and targeted therapies for unresectable pancreatic cancer, are all studied separately from the surgeon's point of view.

## 2. Palliative Surgery and Quality of Life Outcome

Until recently, the term palliative surgery was used to describe a resection with microscopic or gross residual tumor left *in situ* at the end of the operation or a resection done for persistent or recurrent disease after treatment failure. Currently, “palliative surgery” is the term used for all surgical procedures applied with the primary intention of improving quality of life (QOL) by relieving symptoms caused by an advanced disease. This new strict definition is in accordance with the established principles of nonsurgical palliative care and clearly distinguished from “non-curative surgery”, which defines operations with curative intent in asymptomatic patients that result in a non-oncological result. Palliative surgery is common in surgical oncology practice, consisting of 10 to 20 percent of all surgery performed [10].

Since the complication rate for palliative surgery is high and not limited to major procedures, there is currently vital need for a validated instrument for measuring QOL outcome for palliative surgical procedures. The European Organization for Research and Treatment of Cancer (EORTC) QLQ-C30 Core Module and Functional Assessment of Cancer Therapy (FACT) have been used for surgical patients, but have not been adapted and prospectively evaluated for the extensive field of palliative surgery [11,12]. The Palliative Surgery Outcome Score (PSOS) is a prospective measure of the impact of palliative surgery, using the absence of a postoperative complication requiring hospitalization, as part of a measurement for QOL following palliative surgery. Due to its simplicity and straightforward outcome, we believe that it should be unanimously applied. It is calculated by using the following equation: Number of Symptom-free, Non-hospitalized Days/Number of Postoperative Days of Life (up to 180 days).

Symptom-free denotes the symptom intended for treatment and free of major complications. Hospitalized denotes days hospitalized for the palliative operation and any additional days to monitor surgical complications or recurrent symptoms. A PSOS value of 0.7 was identified by patients and families who had good to excellent palliation as an acceptable outcome score [10].

## 3. Obstructive Jaundice

Obstructive jaundice is present in approximately 90% of patients with pancreatic cancer at the time of diagnosis. Pruritus is the most tormenting symptom and liver dysfunction and hepatic failure—secondary to bile stasis and cholangitis—are the most severe consequences. Relief of obstructive jaundice should always be a major therapeutic goal, since it results in a substantial positive impact in the quality of life of these patients [13]. Endoscopic biliary stent placement and a surgical biliary bypass are the two main therapeutic alternatives. The introduction of self-expandable metal endoprostheses significantly reduced the frequency of stent occlusion, which is reported as the main complication of plastic stent placement [14]. Furthermore, covered self-expandable metal stents have been proven superior to the uncovered ones, in terms of tumor ingrowth prevention, though attention must be paid for complications such as acute cholecystitis and pancreatitis [15]. Nevertheless, the success rates for short-term relief are comparable for endoscopical and surgical biliary drainage procedures and range from 80% to 100%. Endoscopy with stent placement seems to lead to shorter hospitalization and lower overall cost, without differentiating in prognosis. However, endoscopic stenting may be associated with fewer early complications and surgical bypass with fewer late complications, making the latter probably a better option for patients more likely to survive longer [16].

Different surgical procedures are available to obtain adequate biliary drainage. External biliary drainage by a T-tube inserted above the site of obstruction has been used in the past, but loss of appetite and electrolyte imbalances are frequent and the technique has been overall abandoned. Internal biliary drainage is generally preferred and can be performed by cholecystojejunostomy, hepatico(choledocho)jejunostomy or choledochoduodenostomy [17]. An extensive review and a randomized controlled trial both showed that choledochojejunostomy, though technically more difficult, has higher success rates to relieve obstructive jaundice and lower rates of recurrent jaundice and cholangitis, compared with cholecystojejunostomy [17,18]. A choledochoduodenostomy is generally not recommended, since it frequently results in recurrent jaundice, due to local tumor growth into the duodenum and the distal common bile duct, encompassing the entrance of the cystic duct.

The choledochojejunostomy is performed by removing the gallbladder and circumferentially dissecting the common hepatic duct, near the bifurcation, and dividing it. The anastomosis can then be performed to a loop of jejunum with a Braun jejunojejunostomy or to a Roux-en-Y limb. In only few patients palliated with a choledochojejunostomy, recurrent jaundice develops before they die of their disease [19].

## 4. Gastric Outlet Obstruction

Whereas, at the time of diagnosis only about 19% of pancreatic cancer patients present with symptoms of gastroduodenal obstruction, such as nausea and vomiting, 30–50% of all patients will eventually develop malignant gastric outlet obstruction (GOO) during the course of their disease, with the necessity for endoscopic or surgical intervention [20]. In order to achieve optimal palliative treatment, it is important to determine the origin of these symptoms. Motility dysfunction of the stomach and duodenum (due to tumor infiltration of the celiac nerve plexus), and mechanical obstruction of the duodenum (resulting from direct tumor ingrowth into the duodenum or from external compression of the duodenum by an adjacent tumor), are two, completely different, entities causing the exact same symptoms [21].

Surgical palliation should be applied only when GOO has a mechanical cause, confirmed by radiological or endoscopical means. At presentation, mechanical obstruction is reported in only around 5% of patients with pancreatic cancer. It is estimated that 3% to 20% of these patients will eventually develop mechanical GOO [22,23]. During laparotomy, in patients who are found to have an unresectable tumor, a gastrojejunostomy can be performed easily, coupled or not with a biliary bypass. Endoscopic duodenal stenting, with self-expandable metallic stents, is another, rather promising therapeutic alternative, according to its early results, for which none solid comparative data (*vs.* surgical treatment) are available at the moment [24,25].

Another issue of great importance and ongoing debate among surgeons is whether a prophylactic gastrojejunostomy should be performed. It must be noted that in only 40–80% of patients with pancreatic cancer, who undergo an exploratory laparotomy, a radical resection is finally performed [5,9,19]. Two randomized controlled trials studied the role of prophylactic gastrojejunostomy, during exploratory laparotomy for unresectable pancreatic cancer [23,26]. No increase of the procedure-related mortality and morbidity rates was noted as well as no differences in hospital stay and survival. None of the patients who received a prophylactic gastrojejunostomy developed GOO later on, compared with 19% of patients who did not undergo a gastrojejunostomy in the initial procedure, the fact that stood out as the main difference. Both trials concluded that a prophylactic gastrojejunostomy may be preferable to a biliary bypass alone, since both tasks proved the same safety, but substantially reduced risk of late GOO. Additionally, several studies show that if performed separately—when the patient presents with symptomatic gastroduodenal stenosis—the morbidity of this second intervention is high, with mortality rates reaching 22% [17,27]. In conclusion, a conventional double bypass procedure, even in the absence of clinically present GOO, may be the golden standard in patients with intraoperatively proven unresectable pancreatic cancer.

## 5. Pain Management

At the time of diagnosis, approximately 40-80% of patients already report pain as one of their main symptoms. During disease progression, almost all patients will have to deal with moderate to severe pain [5]. The pain of pancreatic cancer is typically located in the epigastrium, radiating to the back (band-like) and generally is caused by tumor infiltration of the mesenteric or celiac nerve plexus. According to the WHO guidelines the initial pain management should be pharmacologic, consisting of analgesics such as Non-Steroidal Anti-Inflammatory Drugs (NSAIDs) and/or oral or transdermic narcotic drugs [28]. The appropriate initial line of treatment is long-acting narcotics enforced by short-acting preparations for breakthrough pain. A key principle is to balance pain control against oversedation in order to maintain both comfortable and functional living. For patients who suffer from postprandial pain, multidisciplinary support is necessary. Postprandial pain may be alleviated by pancreatic enzyme replacement therapy (PERT) coupled with a proton-pump inhibitor [29].

The next therapeutic step is a celiac plexus nerve block, as was first described by Kappis in 1914. This relatively simple procedure can be applied percutaneously, but may cause side effects, like diarrhea and orthostatic hypotension, in approximately 40% of patients [30]. When performed percutaneously, an analgesic, such as lidocaine and an anti-infammatory or a neurolytic agent, is injected into the celiac plexus. At some centers, this procedure may be done endoscopically with endoscopic ultrasound guidance, but it can also accomplished by fluoroscopic and CT guidance. The approach may be made from either an anterior or a posterior approach, based on anatomy and the patient's comfort.

Neurolytic celiac plexus blockade (NCPB) may also be applied intraoperatively, on initial surgical exploration, with 50% ethanol or 6% phenol under direct visualization [31]. A double-blind randomized controlled trial showed that intraoperative chemical splanchnicectomy by injection of 20 mL of 50% alcohol on each side of the aorta at the level of the celiac axis *versus* same amount of saline placebo, significantly reduced the mean pain-score for surviving patients, up to six months of follow-up [32]. In addition, significantly more patients never reported pain until death. The effect was not permanent, since around 10% of patients in both groups required an additional percutaneous celiac axis block later on. Additionally, NCPB was found to decrease the subsequent onset pain, even in patients without preexisting pain at the time of surgery. Finally, the same study showed that actuarial survival was improved in the subgroup of patients who reported significant preoperative pain and underwent an alcohol-splanchnicectomy. These latter findings confirm other reports that the presence of pain is associated with a poor prognosis [33]. Conclusively, intraoperative NCPB, as first introduced in the 1960s, is a rather simple and safe procedure, which seems to lead to complete or partial pain-relief in 90% of patients, however the duration of the response is limited, and as patients live longer, soon systemic analgesics become necessary to re-control pain [30,34].

Radiotherapy also can be applied to reduce pancreatic cancer-related pain, however, it is time-consuming, until proven effective, and it is related with considerable toxicity [35]. Another method to palliate pain is the thoracoscopic splanchnicectomy, applied bilaterally or left-sided unilaterally [36,37]. Both techniques have been proven effective, but the complication rate of bleeding and the consequent need for a rescue-thoracotomy has been reported of up to 10% [36].

As mentioned above, pain in pancreatic cancer is believed to be mainly caused by peri-pancreatic nerval infiltration. However, in some patients a significantly dilated pancreatic duct beyond a stenosis is present, along with aggravation of pain after meals. This type of “obstructive pain”, which has been well documented in chronic pancreatitis [38], seems to contribute substantially to the overall pain sensation of this latter subgroup of pancreatic cancer patients. For the vast majority of these cases, pancreatic duct decompression by endoscopic stent placement is a both feasible and safe modality, which may lead to significant pain relief—up to discontinuation of opioid treatment—and short-term improvement of the quality of life [39,40].

In accordance with the available data, a chemical splanchnicectomy during the initial double bypass procedure for unresectable pancreatic cancer must be performed. In all other patients, pharmacologic pain management should be the first therapeutic step. For recurrent pain in all cases, percutaneous or ultrasound-guided celiac plexus block is considered the treatment of choice.

## 6. Palliative Pancreatic Resection

Over the last three decades, specialized centers have managed to increase the overall resection rate in patients with pancreatic cancer to 40–50%, and at the same time to decrease the perioperative mortality rate to 1–2%. Nevertheless, only a percentage of these procedures finally meet the criteria of an R0 resection, in terms of complete tumor removal, keeping the overall prognosis at low levels [19,41,42]. Despite major advances in imaging media, surgical exploration in case of non-conclusive diagnosis remains the only way to definitively decide on resectability. Furthermore, the resectability of pancreatic tumors seems to depend highly on the experience of each individual surgeon and the case volume of the hospital [43].

Several studies have explored the indications of a pancreaticoduodenectomy as a palliative treatment option [42,44,45]. This debatable matter has been advocated by recent data showing that morbidity and mortality rates after pancreaticoduodenectomy are substantially decreasing. Late studies state 5% or even zero mortality, compared to 25% mortality rates in earlier reports, mainly due to a hospital volume effect and partly due to improved management of severe complications [5,9,44,46,47].

Once again, it is of critical value to accurately determine “palliative resection”. This term should not be used for R1 resection, meaning macroscopically radical resection, which proved to be microscopically nonradical after pathologic examination. Such a procedure can be considered as “palliative in retrospect”. “Palliative resection” should be used only for R2 resections, meaning resection initially planned to have macroscopically positive margins. It mainly involves situations in which the tumor is found unresectable after a point of no return or resection for preoperative tumor bleeding non-responding to embolization or other conservative methods [9].

There are no prospective studies involving initially planned R2 resection, but results of R1 resection are available. Two retrospective studies examined the role of a pancreaticoduodenectomy for palliation, by comparing outcomes between non-radical resections and single or double bypass procedures for locally advanced pancreatic cancer without evident metastases [42,44]. Results showed that both groups had similar morbidity and mortality rates and hospital stay; however, in the resection group a significantly longer survival was recorded. Confirmative results were also brought from a similar retrospective study, which showed that margin positive pancreaticoduodenectomy is superior to palliative bypass, by substantially increasing the median survival (resection group: 15.6 month *vs.* bypass group: 6.5 months) [48]. Patient selection and the overall limitations of a retrospective study probably explain that difference.

In a recent randomized, multicenter trial, surgical resection was compared with radiochemotherapy alone in patients with resectable and locally invasive pancreatic cancer, without infiltration of major local vessels or distant metastasis [49]. Patients assigned to the resection group underwent pancreaticoduodenectomy or distal pancreatectomy for resection of the pancreatic tumor. In these patients, no postoperative adjuvant treatment was performed unless recurrence was obvious, at which point the selection of another therapy was permitted. In patients assigned to the radiochemotherapy group, the abdomen was closed once a biopsy had been taken to confirm the diagnosis, although the surgeon was free to perform biliary or gastric bypass. After five-year follow up or longer, the surgery group was found to have significantly better survival than the radiochemotherapy group (P < 0.03). Surgery increased the survival time and three-year survival rate by an average of 11.8 months and 20%, respectively, and it halved the instantaneous mortality (hazard) rate.

Palliative resection still remains a controversial topic. The available data demonstrate that on the setting of questionable resectability a pancreatic resection may offer good palliation and that tumor resection should be performed whenever possible, even in patients with advanced pancreatic cancer. Despite the fact that during explorative laparotomy pancreatic cancer very often presents at a stage where R0 resection is unlikely, an aggressive surgical approach to the tumor may stand as an acceptable option to improve patient prognosis [50]. On the other hand, it is unanimously accepted that resections in patients who are found without doubt unresectable at preoperative diagnostic workup, should not be performed.

## 7. Laparoscopic Palliation

Diagnostic laparoscopy has been widely used in the workup for patients with pancreatic tumors. Nevertheless, the improvement of CT techniques has led to the reduction of the benefit of diagnosing metastases laparoscopically. On the other hand, if a tumor is found unresectable during laparoscopy, minimally invasive palliative procedures are proposed, such as cholecystojejunostomy for obstructive jaundice and gastroenterostomy for duodenal obstruction. Several studies show that laparoscopic double bypass can be both effective and safe, though not efficient data on long-term follow-up exist. What needs to be highlighted is that on the setting of a positive diagnostic laparoscopy for unresectable pancreatic cancer, there is no solid evidence for performing a subsequent open palliative procedure. In other words, minimally invasive palliative procedures ought to be compared only with new endoscopic techniques, such as biliary or duodenal stenting.

The role of staging laparoscopy in pancreatic cancer and the consequent decision-making algorithm for the cases found unresectable varies widely among different studies. Some authors support that the routine use of diagnostic laparoscopy prevents unnecessary laparotomies by detecting small intra-abdominal lesions, which are not identified on preoperative imaging workup [51]. As reported, diagnostic laparoscopy will reveal small (<1 cm) peritoneal or liver surface metastases and finally upstage (to Stage IV) approximately 10% of patients with tumors in the pancreatic head, and probably a greater percentage of those with tumors in the body or tail. The addition of laparoscopic ultrasound in doubtful cases after complete preoperative workup may also have a good yield [52]. Others claim that there are no solid data to justify such practice, since all patients who are found to have a resectable tumor at preoperative diagnostic workup should undergo an exploratory laparotomy immediately [53]. Controversy also surrounds the management of patients who are found to have unresectable disease on staging laparoscopy. If the surgeon is familiar with the laparoscopic techniques, laparoscopic gastric outlet obstruction bypass and laparoscopic biliary decompression seem to provide good results compared to open surgical procedures [54]. On the contrary, some prefer not to proceed to any laparoscopic palliative procedure and to treat any potential biliary or duodenal obstructions with endoluminal stents. If endoscopic stent placement fails, then a surgical biliary bypass should be advocated. As for an open gastroenterostomy, it must be reserved for patients who have confirmed duodenal obstruction [22].

## 8. New Era of Neoadjuvant and Targeted Therapies for Unresectable Pancreatic Cancer

The most promising advance in treating locally advanced pancreatic cancer has been the fact that systematic treatment has the potential to downstage tumors and to allow secondary surgical management [55]. The feasibility of downstaging tumors in anticipation of future resection led to a crucial categorization of locally advanced pancreatic cancer into two distinct groups: borderline resectable and unresectable [55,56]. According to the National Comprehensive Cancer Network's definition, borderline resectable pancreatic head (and body) cancer refers to tumor abutment of the superior mesenteric artery (SMA), severe unilateral superior mesenteric vein or portal vein impingement, gastroduodenal artery encasement up to its origin from the hepatic artery, or colon and mesocolon invasion [57]. The M.D. Anderson Cancer Center introduced a more objective approach to resectability evaluation, based on anatomical Computed Tomography (CT) findings [52]. This latter approach widens the definition of borderline resectable pancreatic cancer, mainly by encompassing reconstructable SMA abutment cases (Table 1).

Before these criteria were established, the standard of care for locally advanced disease had been concurrent chemoradiation therapy (CRT) without aiming to a secondary surgical treatment [58,59]. The criteria shown above allow borderline resectable disease with no vascular involvement to be distinguished from locally advanced unresectable disease with vascular involvement. Both these categories may benefit from neoadjuvant therapy, and secondary resection has emerged as a reachable therapeutic goal. Early attempts at preoperative treatment using chemotherapy, radiotherapy, and CRT demonstrated that neoadjuvant treatment of previously inoperable pancreatic tumors may result in potentially curative secondary surgery [60-62]. In this scenario, the main goal of neoadjuvant therapy for borderline resectable patients is to induce a measurable tumor response and, therefore, increase the likelihood of radical resection with negative margins. Additionally, exposure of the tumor to chemotherapeutic agents before resection allows for the sensitivity of the tumor to those agents to be assessed. Tumors that resist therapy or even keep progressing may be those with aggressive biology that would progress, even if resected and treated postoperatively. These patients who progress during neoadjuvant treatment, should therefore be spared the morbidity and mortality of a major open surgical procedure. On the other hand, patients with favorable responses to preoperative therapy, as demonstrated by imaging tumor regression and decrease of serum tumor marker levels, may have the best chance for an R0 resection and a favorable long-term outcome [55].

Previously, trials failed to distinguish borderline resectable from resectable or borderline unresectable disease. In the single study to address this subgroup, a combination of radiosensitizing agents with 50.4 Gy of radiation was tested in 13 patients with borderline resectable disease [63]. All patients underwent secondary open exploration with the intent of an oncological resection. Eighty-five percent (11 patients) had complete, or R0, resections, which led to a two-year survival of 69% (n = 9) and eight patients disease-free at two years.

Although there have been no recent trials comparing adjuvant chemotherapy to CRT for resected pancreatic cancer, this comparison is being made in the setting of locally advanced pancreatic cancer. The Eastern Cooperative Oncology Group study-4201(ECOG-4201) is the first trial that directly compared gemcitabine in combination with radiation therapy *vs.* gemcitabine alone in patients with locally advanced pancreatic cancer. The concurrent CRT was found to be more myelotoxic and was also associated with considerable gastrointestinal side effects and fatigue. However, gemcitabine when coupled with radiation therapy was found to significantly improve overall survival by tripling the survival rate at 24 months for patients with locally advanced pancreatic cancer [64].

Taken together, the trials conducted in locally advanced pancreatic cancer suggest that CRT is not only tolerable but also can downstage the disease, thus making a secondary oncological resection feasible and consequently prolonging survival. The possibility of using full-dose gemcitabine as a radiation sensitizer could be the next big step, but phase III trials have not yet been conducted with this pattern. Until such trials are available, a reasonable standard of care for locally advanced pancreatic cancer is the RTOG 97-04 sandwich approach with full-dose gemcitabine before and after CRT with 5-FU [65].

A number of genetic alterations have been identified to occur in pancreatic cancer. Commonly mutated genes include the oncogenes *K-ras* (75%–100%) *HER2/neu* (about 65%), *p16Ink4a* (90%), *notch1*, *Akt-2*, and *COX-2* and also the tumor suppressor gene *p53* (45%–75%) *DPC4* (approximately 50%), *FHIT* (70%), and *BRCA2*. Despite this diversity of mutations, none of these genes are currently being targeted in clinical practice [66]. The promise of targeted therapies nevertheless continue to hold great interest in this disease, and other approaches, such as concentrating the role of the tumor stroma, overcoming resistance mechanisms to chemotherapy, and recruiting immune defenses, show early promising results.

The tyrosine kinase inhibitor erlotinib is currently the first and only molecularly targeted therapy approved by the FDA for first-line treatment of advanced pancreatic cancer in combination with gemcitabine [67]. A monoclonal antibody against HER1/EGFR, cetuximab, has been demonstrated to be effective in pancreatic cancer when combined with gemcitabine [68]. The vascular epithelial growth factor (VEGF) and its receptors are promising targets for antineoplastic therapy, since they may induce an improved chemotherapy delivery to the tumor. In a phase II trial, the anti-VEGF monoclonal antibody bevacizumab demonstrated activity in advanced pancreatic cancer with a response rate of 21% and a median survival time of 8.8 months [69].

The role of the pancreatic cancer stroma is an area of ongoing research regarding the pathogenesis pathways of the disease and its dominant resistance to chemotherapy [70,71]. Pancreatic adenocarcinoma is characterized by a strong desmoplastic reaction, which may promote the malignant phenotype [72,73]. Pancreatic stellate cells (PSC) have been shown to produce substances that facilitate the invasion of pancreatic cancer. The level of paracrine secreted protein acidic and rich in cysteine (SPARC) from PSC has been demonstrated to be inversely proportional to survival [72]. This makes SPARC in the PSCs an attractive adjunct target. The human equilibrative nucleoside transporter 1 and human concentrative nucleoside transporter 1 and 3 are responsible for gemcitabine uptake into tumor cells. Lack of these transporters denotes a poorer prognosis with adjuvant treatment and predisposes to resistance to therapy [74]. A promising new nucleoside analog that bypasses this mechanism has shown some benefit in refractory solid tumors in phase I trials, including stabilization of the disease in some pancreatic cancer patients.

## 9. Conclusions

Despite all new treatment modalities for pancreatic cancer, survival remains poor. More than 80% of patients are not eligible for cure therapy at the time of diagnosis. The most common problems of these patients are obstructive jaundice, gastric outlet obstruction and pain, and efficient palliation for those is considered as optimal treatment in most of the cases.

Palliative recommendations for obstructive jaundice include a biliary bypass on relatively fit patients, with a relatively long survival. Compared to endoscopic stenting, biliary bypass has a worse initial morbidity, but offers a better long-term prevention of recurrent obstruction. Stents are proposed as the best choice for patients with a 3-6 months survival. When a surgical biliary bypass has been decided, gastric bypass should also be performed routinely. Nevertheless, the introduction of novel techniques with endoscopic duodenal stenting may soon alter this recommendation.

Approximately 90% of patients with pancreatic cancer will eventually suffer from moderate to severe pain, which has a dramatic impact on the quality of life and co-determines probably even survival. Analgesic therapy is recommended as the initial treatment, but unfortunately only in a small minority of the cases it is proven efficient as monotherapy. Neurolytic celiac plexus blockade is a well documented efficient therapy, which can be applied percutaneously or during laparotomy. For the subgroup of patients with “obstructive component of pain”, pancreatic duct decompression by endoscopic stent placement seems to provide excellent results in pain relief. On the setting of a tumor found unresectable during open exploration, chemical splanchnicectomy should be performed. There are no solid data to conclusively validate the role of palliative R2 resection, though many studies suggest aggressive surgical approach, as more efficient than other palliative procedures.

A multidisciplinary approach with CRT holds the promise of downstaging a locally advanced cancer by diminishing the perivascular neoplastic tissue and even distant micrometastatic disease, and results in a survival advantage as compared with unresected patients. Although the optimal regimen has not been identified, there is strong phase II evidence that full-dose gemcitabine can be tolerated in combination with adequate radiation, and this dose is theoretically most likely to address to micrometastatic disease outside of the radiation field. Finally, trials for targeted therapies hold great promise on achieving better response to the existing adjuvant therapies or even producing new effective genetic therapeutic tools.

## Figures and Tables

**Table 1.** CT-based criteria for categorizing a pancreatic cancer as resectable, borderline resectable or unresectable.

	**Resectable**	**Borderline resectable**	**Unresectable**
**Major veins**	Patent SMV and portal vein	Severe SMV impingement or Reconstructable SMV occlusion	Unreconstructable SMV/portal occlusion

**Major arteries**	Clear fat plane around celiac axis and SMA	Less than 180° abutment of SMA	Greater than 180° SMA encasement
		Reconstructable abutment or encasement of the common hepatic artery	Unreconstructable SMA involvement
			Any celiac abutment (head mass)
			Greater than 180° SMA encasement (body mass)

**Aorta**			Aortic invasion or encasement

**Metastases**	No distant metastases		Distant metastases
			Metastases to LN beyond the field of resection

SMV = superior mesenteric vein, SMA = superior mesenteric artery, LN = lymph nodes.
